# Isolation of *Bartonella henselae* and Two New *Bartonella* Subspecies, *Bartonella* *koehlerae* Subspecies *boulouisii* subsp. nov. and *Bartonella koehlerae* Subspecies *bothieri* subsp. nov. from Free-Ranging Californian Mountain Lions and Bobcats

**DOI:** 10.1371/journal.pone.0148299

**Published:** 2016-03-16

**Authors:** Bruno B. Chomel, Sophie Molia, Rickie W. Kasten, Gina M. Borgo, Matthew J. Stuckey, Soichi Maruyama, Chao-chin Chang, Nadia Haddad, Jane E. Koehler

**Affiliations:** 1 Department of Population Health and Reproduction, School of Veterinary Medicine, University of California Davis, Davis, California, United States of America; 2 Centre de Coopération Internationale en Recherche Agronomique pour le Développement, Campus International de Baillarguet, TA C-22/E, Montpellier, France; 3 Microbial Pathogenesis and Host Defense Program, and Division of Infectious Diseases, University of California San Francisco, San Francisco, California, United States of America; 4 Laboratory of Veterinary Public Health, Department of Veterinary Medicine, College of Bioresource Sciences, Nihon University, Tokyo, Japan; 5 Graduate Institute of Microbiology and Public Health, School of Veterinary Medicine, National Chung Hsing University, Taichung, Taiwan; 6 UPE, Ecole Nationale Vétérinaire d'Alfort, UMR-BIPAR, ENVA, ANSES, UPEC, USC INRA, LERPAZ, Maisons Alfort, France; Beijing Institute of Microbiology and Epidemiology, CHINA

## Abstract

Domestic cats are the natural reservoir of *Bartonella henselae*, *B*. *clarridgeiae* and *B*. *koehlerae*. To determine the role of wild felids in the epidemiology of *Bartonella* infections, blood was collected from 14 free-ranging California mountain lions (*Puma concolor*) and 19 bobcats (*Lynx rufus*). *Bartonella* spp. were isolated from four (29%) mountain lions and seven (37%) bobcats. These isolates were characterized using growth characteristics, biochemical reactions, molecular techniques, including PCR-RFLP of selected genes or interspacer region, pulsed-field gel electrophoresis (PFGE), partial sequencing of several genes, and DNA-DNA hybridization. Two isolates were identical to *B*. *henselae* genotype II. All other isolates were distinguished from *B*. *henselae* and *B*. *koehlerae* by PCR-RFLP of the *gltA* gene using endonucleases *Hha*I, *Taq*I and *Aci*I, with the latter two discriminating between the mountain lion and the bobcat isolates. These two novel isolates displayed specific PFGE profiles distinct from *B*. *henselae*, *B*. *koehlerae* and *B*. *clarridgeiae*. Sequences of amplified gene fragments from the three mountain lion and six bobcat isolates were closely related to, but distinct from, *B*. *henselae* and *B*. *koehlerae*. Finally, DNA-DNA hybridization studies demonstrated that the mountain lion and bobcat strains are most closely related to *B*. *koehlerae*. We propose naming the mountain lion isolates *B*. *koehlerae* subsp. *boulouisii* subsp. nov. (type strain: L-42-94), and the bobcat isolates *B*. *koehlerae* subsp. *bothieri* subsp. nov. (type strain: L-17-96), and to emend *B*. *koehlerae* as *B*. *koehlerae* subsp. *koehlerae*. The mode of transmission and the zoonotic potential of these new *Bartonell*a subspecies remain to be determined.

## Introduction

The genus *Bartonella* is comprised of aerobic Gram-negative bacteria. An increasing number of *Bartonella* species and subspecies are being recognized as emerging pathogens [1]. Seventeen of the 33 *Bartonella* species and 3 subspecies currently described are associated with human disease ([2] http://www.bacterio.net/bartonella.htm). The spectrum of diseases caused by *Bartonella* species in humans is expanding, and includes Carrion’s disease, trench fever, cat scratch disease, bacillary angiomatosis, peliosis hepatis, endocarditis, chronic bacteremia, neuroretinitis, encephalitis, and fever of unknown origin [1]. The most common *Bartonella* infection in people is caused by *B*. *henselae*, the etiological agent of cat scratch disease (CSD). Domestic cats (*Felis catus*) are known to be the natural reservoir of *B*. *henselae*, as well as of *B*. *clarridgeiae* and *B*. *koehlerae* [3–7]. *B*. *bovis* has also been isolated from a few domestic cats, and initially was provisionally named *B*. *weissii* by Regnery and colleagues at the Centers for Disease Control in Atlanta, Ga. (R. Regnery, N. Marano, P. Jameson, E. Marston, D. Jones, S. Handley, C. Goldsmith, and C. Greene, 15th Meet. Am. Soc. Rickettsiol., 2000, abstr. 4). Very rarely, *B*. *quintana* and *B*. *vinsonii* subsp. *berkhoffii* have been isolated from or detected in domestic cats [8, 9].

Many free-ranging wild felids live in North America. An estimated 5,100 mountain lions (*Puma concolor*), also known as pumas or cougars [10] and an equal or larger number of bobcats (*Lynx rufus*) (P. Swift, unpublished data) live in California. Overlapping habitats can lead to the spread of ectoparasites between wild and free-ranging domestic cats. This interface is epidemiologically important because the majority of *Bartonella* spp. are vectored by arthropods that could potentially facilitate transmission between felid species and humans.

In previous studies in California, 26 (35%) of 74 free-ranging mountain lions and 33 (53%) of 62 free-ranging bobcats were seropositive for *B*. *henselae* [11]. A serological survey of vector-borne zoonoses in 442 mountain lions during the period 1987–2010 revealed a seroprevalence of 37.1% for *B*. *henselae*, with the highest exposure in central coastal California [12]. DNA amplified from the blood of three of seven mountain lions, which were strongly seropositive for *Bartonella*, was genetically similar to *B*. *henselae* [12]. Another study reported a seroprevalence of 16% in mountain lions and 31% in bobcats from southern California and Colorado [13]. In Florida, antibodies against *B*. *henselae* were also detected in two (28%) mountain lions originating from Texas (*Puma concolor stanleyana*), and in five (18%) Florida panthers (*Puma concolor coryi*) [14]. In a larger study of 479 mountain lions and 91 bobcats from North America, Central America, and South America, the overall prevalence of *B*. *henselae* antibodies was 19.4% in mountain lions and 23.1% in bobcats [15]. In the United States, seroprevalence of *B*. *henselae* in mountain lions from the southwestern region was found to be almost three times higher than in mountain lions from the northwestern and mountain states [15]. Because systematic culture, isolation and PCR-based speciation of *Bartonella* strains were not performed in previous studies, it is not known whether the *Bartonella* strains in wild felids, which have the potential for transmission from wild felids to domestic cats or vice-versa, are distinct from those in domestic cats. The objective of this study is to describe the *Bartonella* species isolated from free-ranging mountain lions and bobcats from California.

## Materials and Methods

### Bacterial strains

*B*. *henselae* genotype I (Houston 1) type strain ATCC 49882 and *B*. *clarridgeiae* ATCC 51734 were obtained from the American Type Culture Collection (Rockville, MD). Strain U4, originally isolated from a naturally infected cat at the University of California, Davis, was used for the *Bartonella henselae* genotype II (Marseille genotype) strain. *B*. *koehlerae* type strain C29 (ATCC 700693) was isolated from a naturally infected barn cat in northern California [4]. *B*. *bovis* (previously *Candidatus* B. weissii), isolated from domestic cats, was kindly provided by Russell Regnery, Centers for Disease Control and Prevention (CDC), Atlanta, Georgia, USA. For DNA-DNA hybridization studies, additional *Bartonella* strains at the CDC included: *B*. *quintana* type strain ATCC VR-358, *B*. *henselae* 88–712, *B*. *henselae* G6486, *B*. *henselae* G6529, *B*. *henselae* G8378, *B*. *henselae* G5691, *B*. *elizabethae* type strain ATCC 49927, *B*. *vinsonii* type strain ATCC VR-152, *B*. *vinsonii* subsp. *berkhoffii* type strain ATCC 51672, *B*. *clarridgeiae* strain “Big Blackie,” *B*. *bacilliformis* type strain ATCC 35685, and *B*. *bacilliformis* strain KC 584.

### Animals

Whole blood samples were collected for culture of *Bartonella* from 14 free-ranging mountain lions and 19 free-ranging bobcats selected by convenience sampling in California between January 1995 and August 2003. Nine mountain lions were trapped in seven different California counties and kept at the California Department of Fish and Wildlife (CDFW) facility, Rancho Cordova, CA, where blood samples were collected between 1995 and 1998, and five mountain lion blood samples were collected during behavioral studies in San Diego County (Cleveland National Forest) between 2001 and 2003 (Table 1). Similarly, 19 bobcats were trapped in three different counties of the California Coast Range (Table 2). Seven of the 19 bobcats were kept at the CDFW facility, where the blood samples were collected. The animals were sedated using ketamine hydrochloride (Ketaset, Fort Dodge Laboratories, Fort Dodge, Iowa 50501, USA) (11 mg/ kg). Four mountain lions and three bobcats housed at the CDFW facility had blood samples collected for bacterial isolation more than once (Tables 1 and 2). The animal use and care protocols were approved by the California Department of Fish and Wildlife (Dr. Pamela Swift) and followed during collection of blood from these animals. We operated under Protocol 10950/PHS, Animal Welfare Assurance number A3433-01, with capture and sampling procedures approved in Protocol number 17233 by the Animal Care and Use Committee at the University of California, Davis, and Memoranda of Understanding and Scientific Collecting Permits from the California Department of Fish and Wildlife (CDFW).

**Table 1:** Descriptive data for 14 California mountain lions (*Puma concolor*) tested between January 1995 and August 2003.

Animal ID	Sex	Age	County	Capture date	Collection date	CFU/ml	IFA* titer
**L-45a-94**	male	6 mo	Butte	10/25/94	01/30/95	0	1,024
**L-45b-94**	male	6 mo	Butte	10/25/94	01/30/95	0	256
**L-42-94**	male	8 mo	Humboldt	09/06/94	01/30/95	3,200	128
					04/03/95	600	±64
					05/03/95	60	<32
**L-27-96**	male	adult	Butte	06/19/96	06/28/96	1,860	256
					07/03/06	1,730	256
**L-07-97**	male	5 yrs	Placer	03/09/97	03/10/97	0	256
**L-14-97**	male	adult	Lake	04/08/97	05/14/97	0	512
**L-39-97**	female	8 wks	El Dorado	10/10/97	10/15/97	0	0
					04/10/98	200	64
					05/21/98	260	64
					10/21/98	300	±64
**L-23-98**	male	11 mo	Monterey	10/97	04/10/98	0	0
					10/21/98	0	0
**FM98061**	female	kitten	Amador	11/98	04/29/99	3,000	256
**F14**	female	5 yrs	San Diego	02/03	02/03	0	0
**F15**	female	2 yrs	San Diego	02/03	02/03	0	0
**F21**	female	< 2 yrs	San Diego	02/03	02/03	0	0
**F18**	female	adult	San Diego	08/03	08/03	0	0
**M16**	male	4 yrs	San Diego	08/03	08/03	0	0

* IFA, indirect immunofluorescence antibody for *B*. *henselae*

**Table 2:** Descriptive data for 19 California bobcats (*Lynx rufus*) tested between May 1995 and September 1998.

Animal ID	Sex	Age	County	Capture date	Collection date	CFU/ml	IFA* titer
**L-12-95**	male	2 yrs	Santa Clara	04/13/95	05/03/95	0	128
**FB95002**	female	1 yr	Santa Clara	04/26/95	05/03/95	0	0
**BC025**	male	1 yr	Marin	06/05/95	06/06/95	0	64
**L-08-96**	female	adult	Santa Clara	04/96	05/16/96	40	128
					06/12/96	570	128
					07/01/96	185	64
**L-13-96**	male	adult	Santa Clara		05/16/96	0	32
					06/12/96	0	64
**L-17-96**	female	adult	Santa Clara		05/16/96	40	128
					06/12/96	1,235	128
**DS03**	female	adult	San Mateo	09/24/96	09/25/96	0	32
**DS07**	male	6 mo.	San Mateo	09/26/96	09/27/96	0	0
**DS08**	male	4 yrs	San Mateo	09/26/96	09/27/96	5	64
**DS11**	male	6 yrs	San Mateo	10/07/96	10/08/96	0	64
**DS13**	male	2 yrs	San Mateo	11/14/96	11/15/96	0	±32
**L-10-97**	male	adult	Santa Clara	03/14/97	05/14/97	21	256
**L-11-97**	female	adult	Santa Clara	03/14/97	05/14/97	40	128
**SC414**	female	5 yrs	Santa Clara	07/29/97	07/29/97	0	32
**SC443**	female	2 mo.	Santa Clara	10/09/97	10/09/97	400	64
**DS488**	male	1 yr	Santa Clara	07/28/98	07/28/98	0	64
**DS491**	female	6 yrs	Santa Clara	07/31/98	07/31/98	0	512
**DS507**	male	6 yrs	Santa Clara	09/09/98	09/09/98	171	64
**DS517**	male	5 yrs	Santa Clara	09/17/98	09/17/98	0	32

* IFA, indirect immunofluorescence antibody for *B*. *henselae*

### Blood Sample Collection

All blood samples (1.5 to 2.0 ml per tube) were collected in pediatric lysis-centrifugation tubes (Wampole Laboratories, Cranbury, N.J.) or plastic EDTA tubes (Becton Dickinson, Franklin Lakes, N.J.). Whenever possible, additional serum samples were collected simultaneously from mountain lions and bobcats into serum separating tubes, to assay for *Bartonella*-specific antibodies. Samples collected in pediatric lysis-centrifugation tubes were brought to the laboratory the same day, stored at 4°C and processed within 48 hours. The samples collected in EDTA tubes were stored either at -70°C or -20°C for one to two weeks, and the serum samples at -20°C, until they were processed.

### Blood culture

The whole blood samples (approximately 1.5 to 2.0 ml) were centrifuged at 5000 x g for 30 min at room temperature (after thawing blood samples stored frozen in plastic EDTA tubes). Blood pellets were resuspended in 125 μl of M199S inoculation medium [5] and plated onto fresh (< 8 days old) heart infusion agar (Difco laboratories, Detroit, MI) containing 5% fresh rabbit blood (HIAR). The plates were then incubated at 35°C in 5% CO_2_ for four weeks, and cultures were examined at least twice weekly for bacterial growth. The number of colonies observed was recorded as the number of colony-forming units per milliliter (CFU/ml) of blood. Colonies were sub-cultured, harvested, and frozen at -70°C.

### Microscopic and biochemical analyses

Gram’s staining and biochemical tests were performed on all isolates. Sterile swabs moistened with filter-sterilized, phosphate-buffered saline (PBS) were used to remove colonies from sub-cultured agar plates to glass slides. Following heat fixation, Gram’s staining was performed, and bacteria were visualized by light microscopy. The motility of cells suspended in heart infusion broth was determined with a 100x oil immersion objective. Standard methods were used to test for select preformed enzymes and carbohydrate utilization [16]. Preformed bacterial enzyme activity was tested using the MicroScan Rapid Anaerobe ID Panel (Dade International Inc., West Sacramento, CA, USA), as previously reported [17].

### Indirect immunofluorescence antibody (IFA) test

Antibody titers against *B*. *henselae* were determined using an IFA test [18]. For antigen preparation, *B*. *henselae* strain U4 (U. C. Davis), a 16 S rRNA genotype II strain originally isolated from a naturally infected cat at the University of California, Davis, was cultivated with *Felis catus* whole fetus (FCWF) cells in tissue culture media for 3–5 days. Similarly, one of the mountain lion isolates (isolate L-42-94) was used as the antigen and was cultivated on the same cell line for 3–5 days. Infected FCWF cells were applied to each well of multi-well, super-cured heavy teflon coated slides (CelLine Associates, Inc., Neufield, NJ) and incubated for 24 hours to allow the cells to adhere to the slides. Slides were then rinsed in phosphate-buffered saline (PBS, pH 7.4), air-dried, acetone fixed, and stored at -20°C after air-drying a final time. Wildcat sera were diluted 1:64 in PBS with 5% skim milk, and added to the test wells of the slides. After washing in PBS, fluorescein-labeled, goat anti-cat IgG (Cappel, Organon Teknika Corp., Durham, NC) was used as the conjugate. Positive and negative controls were included on each test slide. Intensity of fluorescence of the bacteria was graded subjectively on a scale of 0 to 4. Fluorescence intensity ≥ 2 at a dilution of 1:64 was considered to be a qualitatively positive result, as previously described [18]. For a quantitative result, positive sera were serially diluted (two-fold dilutions) to obtain an endpoint titer. Reading of the slides was performed independently by two readers for all serum samples.

### DNA extraction and PCR

Sub-cultured colonies were scraped off agar plates and suspended in 100 μl of sterile water. The bacterial suspension was heated 15 min at 100°C and centrifuged at 15,000 × g for 10 min at 4°C [19]. The supernatant, diluted 1:10, was then used as a template for amplification of the *gltA*, 16S rRNA gene, *ftsZ*, *rpoB*, *ribC* and *groEL* genes, and the 16S-23S intergenic spacer region (ITS) for either PCR-restriction fragment length polymorphism analysis (RFLP) or sequencing. Approximately 380 base pairs (bp) of the *gltA* gene [20], 1,500 bp of the 16S rRNA gene [6, 21], 900 bp of the *ftsZ* gene [22], 825 bp of the *rpoB* gene [23], 580 bp of the *ribC* gene [24], 1,500 bp of the *groEL* gene [25] and fragments of various sizes of the 16S-23S ITS [26] were amplified using previously described primers and methods, and verified by gel electrophoresis.

### PCR-RFLP analysis

The amplified products were enzymatically digested overnight using the appropriate restriction endonucleases. The amplified product of the *gltA* gene was digested with *Taq*I (Promega, Madison, WI), *Hha*I (New England Biolabs, Beverly, M.A.), *Mse*I (New England Biolabs) and *Aci*I (New England Biolabs); the amplified product of the 16S rRNA gene was digested with *Dde*I (New England Biolabs); the amplified product of the *ribC* gene was digested with *Taq*I; and the amplified product of the 16S-23S ITS region was digested with *Taq*I and *Hae*III. The digestion temperature was 65°C for *Taq*I and 37°C for all other enzymes. The digested fragments were separated by electrophoresis in a 3% Nusieve GTG agarose gel (Biowhittaker Molecular Applications, Rockland, ME). Fragment sizes were estimated by comparison with a 100 bp ladder (Invitrogen, Carlsbad, CA). Control samples included DNA from a strain isolated from a naturally-infected cat, confirmed previously to be *B*. *henselae* by 16S rRNA gene sequencing, and a negative control sample with no DNA template. PCR-RFLP profiles of isolates were compared with *B*. *henselae* genotype I and genotype II, *B*. *koehlerae*, *B*. *clarridgeiae* and *B*. *bovis*.

### Pulsed field gel electrophoresis (PFGE)

For PFGE, a single colony of each isolate was sub-cultured to confluence (in a total of one or two passages) on HIAR at 35°C for 5–7 days in a 5% CO_2_ atmosphere. Bacteria grown on the agar plates were scraped off, suspended in sterile saline, and washed twice by centrifugation at 15,000 x *g* for 5 min at 4°C. The turbidity of the suspension was adjusted to McFarland standard 6 [19], and 0.5 mL of this suspension was mixed gently but thoroughly with an equal volume of 2% ultrapure low-melting-point agarose (Gibco BRL Life Technologies). The mixture was solidified in plug molds at 4°C, and the agarose plugs were transferred into lysozyme solution (10 m*M* Tris [pH 7.2], 50 m*M* NaCl, 0.2% sodium deoxycholate, 0.5% sodium lauroyl sarcosine, and 1 mg/mL lysozyme) and incubated at 37°C overnight. The plugs were rinsed with sterile water and incubated in proteinase K solution (100 m*M* EDTA [pH 8.0], 0.2% sodium deoxycholate, 1% sodium lauroyl sarcosine, and 1 mg/mL proteinase K) at 50°C overnight. The proteinase K incubation was repeated a second time. The plugs then were washed four times in 10 mL of washing buffer (50 m*M* EDTA and 20 m*M* Tris [pH 8.0]) for 1 h at room temperature with gentle agitation. Proteinase K was inactivated by the addition of 1 m*M* phenyl-methyl-sulfonyl-fluoride solution during the second wash. The plugs were stored in wash buffer at 4°C before endonuclease digestion.

Before digestion, the plugs were transferred to 1.5-mL sterile microtubes with 0.1× wash buffer at 4°C overnight and then equilibrated in 1× endonuclease-specific reaction buffer for 1 h. *Sma*I restriction endonuclease (New England BioLabs) was used for the analysis of total *Bartonella* genomic DNA from the different species and isolates by digesting bacterial chromosomal DNA in reaction buffer at 28°C overnight. After digestion, plugs were equilibrated in 0.5× TBE buffer (45 m*M* Tris-borate and 1 m*M* EDTA [pH 8.0]) for 30 min. The chromosomal restriction fragments were separated by PFGE in a CHEF-DRIII system (Bio-Rad) using a 1.5% pulsed-field certified agarose gel (Bio-Rad) in 0.5× TBE buffer. The electrophoresis was equilibrated at 14°C for 26 h at a constant voltage of 5.7 V/cm for the *Sma*I-digested plugs. Separation of the digested genomic DNA in plugs was achieved with pulse times of 3–10s. After electrophoresis, the gel was stained and photographed. Lambda ladder pulsed-field gel marker (48.5–970 kbp; Bio-Rad) was used for molecular weight standards. *B*. *henselae* Houston-1 (ATCC 49882) was always included as a positive control for assurance of the consistent performance of the digestion and electrophoresis conditions [19].

### Sequencing of gene fragments for phylogenetic analysis

PCR products used for DNA sequencing were purified with QIAquick PCR purification kit (QIAGEN Sciences, Maryland) and sequencing was done using a fluorescence-based automated sequencing system (Retrogen Sequencing, San Diego, CA). For the phylogenetic analysis, forward and reverse direction sequences of the 379 bp *gltA* gene fragment, 935 bp *ftsZ* gene fragment, 893 bp *rpoB* gene fragment, and the various sized fragments (721 bp to 723 bp) of the 16S-23S ITS region were joined to form a concatenated sequence. Sequence data were imported into MEGA version 5.0 software (http://www.megasoftware.net) and aligned by the Clustal W program. A neighbor-joining tree was constructed [27] and bootstrap replicates were performed to estimate node reliability of the phylogenetic tree, with values obtained from 1000 randomly selected samples of the aligned sequence data. The evolutionary distances were computed using the Kimura’s two-parameter method [28].

### DNA-DNA hybridization methods

Mountain lion strain L-42-94 was grown on HIAR plates at 35°C in 5% CO_2_ for 7 days, scraped from the agar surface and washed twice with sterile 0.1M NaCl. The methods used for DNA extraction and purification as well as the hydroxyapatite method have been described previously [29]. DNA was labeled enzymatically *in vitro* with [alpha-^32^P] dCTP by use of a nick translation reagent kit (GIBCO BRL, Gaithersburg, Md.), according to the manufacturer's instructions. Divergence in related sequences was estimated to approximately 1% for each degree of decreased thermal stability in a heterologous re-associated DNA duplex, compared with that in the homologous re-associated DNA duplex [29]. Calculation of divergence was to the nearest 0.5%.

## Results

### Primary isolation of *Bartonella* species

During primary isolation, very small colonies were observed growing on HIAR plates after a minimum of 14 days following plating of the pelleted blood from four (29%) of the 14 mountain lions (Table 1) and seven (37%) of the 19 bobcats (Table 2). None of the five mountain lions from southern California had detectable bacteremia, whereas the prevalence of bacteremia was 44% (4/9) in the mountain lions from northern and central California. All bacterial colonies were homogeneous, round, grey-white in color, 0.3 to 1.0 mm in diameter and embedded in the medium, and either rough, such as for mountain lion isolate L-42-94, or smooth for bobcat isolates L-10-97, L-11-97.

Each of the three blood samples collected from mountain lion L-42-94 between January 30th, 1995 and May 3rd, 1995 yielded organisms (range: 3,200 CFU/ml in January to 60 CFU/ml in May 1995) (Table 1). Similarly, the second bacteremic mountain lion (L-27-96) had a high level of bacteremia (1,730 to 1,860 CFU/ml of blood). The third culture-positive mountain lion (L-39-97) was a young female that had been captured at eight weeks of age, at which time it was serologically negative and culture negative. This mountain lion was kept in a cage in an enclosed area. When re-tested at eight, nine and 14 months of age, the animal was bacteremic and seropositive (Table 1). The last isolate (FM98061) was from a female mountain lion kitten that was highly bacteremic (3,000 CFU/ml). We were able to document bacteremia in some of these wild felids for at least six months. In the seven bacteremic bobcats, the level of bacteremia varied from 5 CFU/ml to more than 1,000 CFU/ml (Table 2).

### Microscopic and biochemical analysis

The phenotypic characteristics of the four mountain lion and seven bobcat strains were compared with *B*. *henselae*, *B*. *koehlerae*, and *B*. *clarridgeiae* (Table 3). Microscopic examination of the bacteria isolated from blood revealed short, slender Gram-negative rods. No motility was detected. Tests for catalase and urease activity were negative. By measuring preformed enzymes (Rapid Anaerobe ID Panel profile number 10477640), the strains were found to be biochemically inert except for the production of peptidases. The preformed enzyme score reading was 10077640 for three of the four mountain lion isolates and 10477640 for six of seven of the bobcat isolates. In contrast, the scores for *B*. *henselae* ATCC 49882 and *B*. *koehlerae* ATCC 700693 were 00077640 and 10073240, respectively [4]. For one bobcat and one mountain lion isolate, the preformed enzyme profile was identical to that of *B*. *henselae*.

**Table 3:** Phenotypic characteristics of *Bartonella* species isolated from domestic and wild felids, including the new subspecies *B*. *koehlerae* subsp. *boulouisii* and *B*. *koehlerae* subsp. *bothieri*.

Characteristic	*B*. *clarridgeiae*	*B*. *henselae*	*B*. *koehlerae*	*B*. *k*. *boulouisii*	*B*. *k*. *bothieri*
Gram reaction	-	-	-	-	-
Oxidase, catalase	-	-	-	-	-
Growth on HIAR*	+	+	-**	+	+
first colonies appeared	<8 days	<8 days	≥14 days	≥14 days	≥14 days
Hemolysis	-	-	-	-	-
Motility	-	-	-	-	-
Indole production	-	-	-	-	-
Urea	-	-	-	-	-
Arginine hydrolysis	+	+	+	+	+
Glycyl-glycine hydrolysis	+	+	+	+	+
Glycine hydrolysis	+	+	+	+	+
Leucine hydrolysis	+	+	+	+	+
Lysine hydrolysis	+	+	-	+	+
Methionine hydrolysis	+	+	+	+	+
Proline hydrolysis	+/-	+	-	+/-	+/-
Tryptophan hydrolysis	+	+	+	+	+
*p*- & *bis-p*-NPP† hydrol.	-	-	-	+/-	+/-

* HIAR: heart infusion agar with 5% rabbit blood

** Grows on chocolate agar

^†^ NPP: Nitrophenylphosphate

### Serology results of samples from bobcats and mountain lions

All bacteremic bobcats and mountain lions were seropositive (titer ≥1:64), using an IFA with *B*. *henselae* genotype II antigen (strain U4, U.C. Davis). In addition, four of the 10 non-bacteremic mountain lions and six of the 12 non-bacteremic bobcats were seropositive (Tables 1 and 2). For mountain lion L-42-94, a decrease of the antibody titer was concomitant with a decline in the level of bacteremia (Table 1). IFA titers were identical or very close (within one dilution factor) when using either *B*. *henselae* or L-42-94 strain as the antigen (data not shown).

### PCR/RFLP analysis

**Fig 1:**
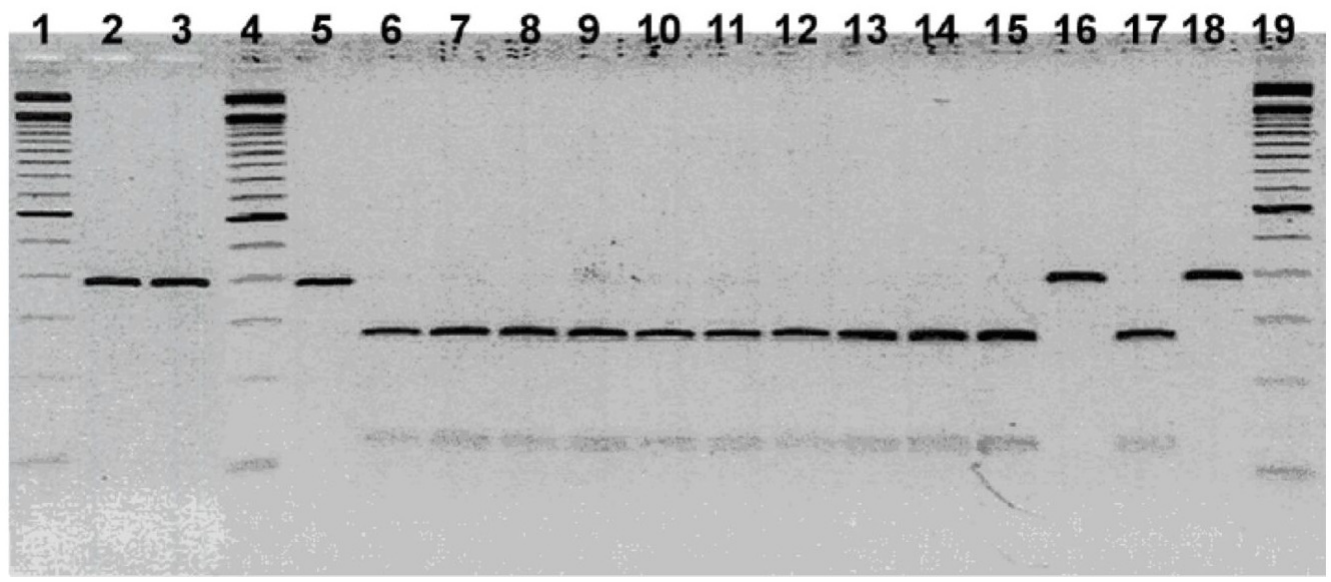
PCR-RFLP of a *gltA* gene fragment using the restriction endonuclease *Aci*I. Lanes 1, 4 and 19 show 100 BP Ladder; Lane 2, Mt Lion L27-96; Lane 3, Mt Lion L42-94; Lane 5, Mt Lion L-39-97; Lane 6, Mt Lion FM98061; Lane 7, Bobcat L08-96; Lane 8, Bobcat L17-96; Lane 9, Bobcat DS08; Lane 10, Bobcat L10-97; Lane 11, Bobcat L11-97; Lane 12, Bobcat SC443; lane 13, Bobcat DS507; Lane 14, *B*. *henselae* Type I; Lane 15, *B*. *henselae* Type II; Lane 16, *B*. *clarridgeiae*; Lane 17, *B*. *koehlerae*; Lane18, *B*. *bovis* (“weissii” isolate).

For the *gltA* gene, all mountain lion and bobcat isolates showed a profile similar to that of *B*. *henselae* genotype I and genotype II after digestion with *Hha*I (Table 4). Digestion of the *gltA* gene with *Mse*I was not discriminatory, as it shared a similar profile with both *B*. *henselae* genotype I and genotype II and *B*. *koehlerae*. However, digestion with *Taq*I identified a profile similar to *B*. *koehlerae* for three of the four mountain lion isolates and six of the seven bobcat isolates. When using *Aci*I endonuclease, a distinction could be made between three of the four mountain lion isolates with a profile similar to *B*. *clarridgeiae*, whereas the profile of all seven bobcats and the fourth mountain lion was similar to *B*. *henselae* and *B*. *koehlerae* (Table 4, Fig 1). One bobcat isolate (SC443) and one mountain lion isolate (FM98061) showed a profile similar to *B*. *henselae* whether using *Taq*I, *Hha*I, *Mse*I or *Aci*I (Table 4). For the 16S rRNA gene digested with *Dde*I, all mountain lion and bobcat isolates had a profile similar to *B*. *henselae* genotype II (Table 5). For the *ribC* gene, the profile was similar to *B*. *henselae* genotype I and genotype II when digesting with *Taq*I endonuclease, and the profile was similar to *B*. *henselae* genotype II for the 16S-23S ITS digested with endonucleases *Taq*I and *Hae*III (Table 5).

**Table 4:** PCR-RFLP comparison of the citrate synthase gene (*gltA*) of two novel *Bartonella* subspecies isolated from California mountain lions and bobcats, with *Bartonella* species isolated from domestic cats: *B*. *henselae* genotype I (Bh I) and genotype II (Bh II), *B*. *koehlerae* (Bk), and *B*. *clarridgeiae* (Bc).

	*Taq*I			*Hha*I			*Mse*I			*Aci*I			
Source of isolate tested	Bh I	Bh II	Bk	Bh I	Bh II	Bk	Bh I	Bh II	Bk	Bh I	Bh II	Bk	Bc
**Mountain lions:**													
** (L42-94, L27-96, L-39-97)**	**-**	**-**	**+**	**+**	**+**	**-**	**+**	**+**	**+**	**-**	**-**	**-**	**+**
** FM98061 (*B*. *henselae*)**	**+**	**+**	**-**	**+**	**+**	**-**	**+**	**+**	**+**	**+**	**+**	**+**	**-**
**Bobcats:**													
** (L08-96, L17-96, DS08, L10- 97, L11-97, DS507)**	**-**	**-**	**+**	**+**	**+**	**-**	**+**	**+**	**+**	**+**	**+**	**+**	**-**
** Sc443 (*B*. *henselae*)**	**+**	**+**	**-**	**+**	**+**	**-**	**+**	**+**	**+**	**+**	**+**	**+**	**-**

**Table 5:** PCR-RFLP comparison of the 16S rRNA gene, *ribC* and internal transcribed spacer region (ITS) of two novel *Bartonella* subspecies isolated from California mountain lions and bobcats, with *Bartonella* species isolated from domestic cats: *B*. *henselae* genotype I (Bh I) and genotype II (Bh II), and *B*. *koehlerae* (Bk).

	16S rDNA			*RibC*			ITS			ITS		
	*Dde*I			*Taq*I			*Taq*I			*Hae*III		
	Bh I	Bh II	Bk	Bh I	Bh II	Bk	Bh I	Bh II	Bk	Bh I	Bh II	Bk
**Mountain Lions**	**-**	**+**	**-**	**+**	**+**	**-**	**-**	**+**	**-**	**-**	**+**	**-**
**Bobcats**	**-**	**+**	**-**	**+**	**+**	**-**	**-**	**+**	**-**	**-**	**+**	**-**

### Pulsed field Gel Electrophoresis (PFGE)

**Fig 2:**
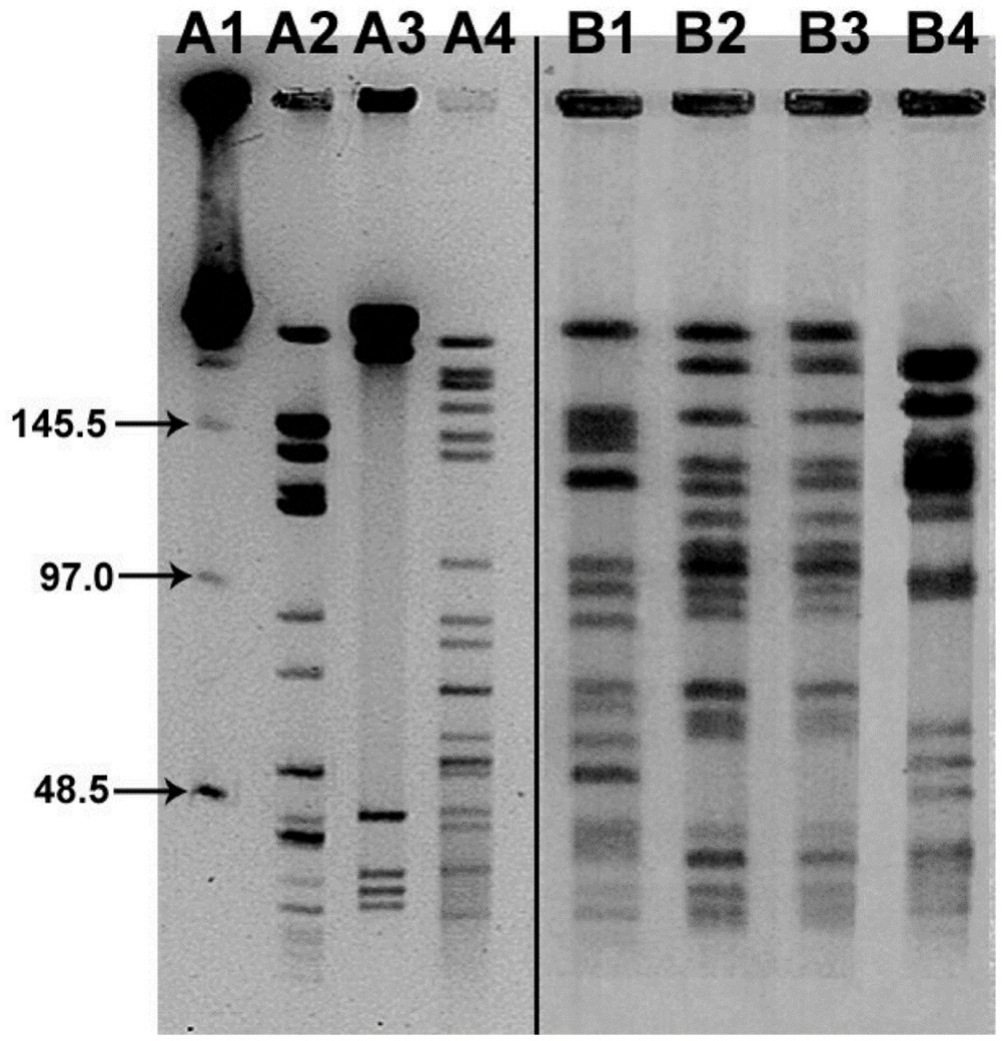
PFGE analysis of *Sma*I-digested genomic DNA from selected *Bartonella* species. Lane A1, molecular size standards (48.5 to 970 kbp); lane A2, *B*. *henselae* (type I Houston-I strain; ATCC 49882); lane A3, *B*. *clarridgeiae* (ATCC 51734); lane A4, *B*. *koehlerae* (ATCC 700693) (from reference 41); lane B1, *B*. *henselae* (type II Marseille strain; U4 U.C. Davis); lane B2, Mt Lion strain L42-94; lane B3, Mt Lion L27-96; lane B4, Bobcat strain L-17-96 (this study).

Two mountain lion isolates (L-42-94 and L-27-96) displayed the same, very distinct and specific bands, distinguishing them from *B*. *henselae* genotype I and genotype II, and also from *B*. *koehlerae* (Fig 2). Additionally, two selected bobcat isolates (L-17-96; and L-08-96) displayed identical, very distinct and specific bands, distinguishing them from *B*. *henselae* genotype I and genotype II, from *B*. *koehlerae*, and from the mountain lion isolates (Fig 2).

### Sequencing of *gltA*, *rpoB* and *ftsZ* gene fragments and a 16S-23S ITS fragment for phylogenetic analysis

**Fig 3:**
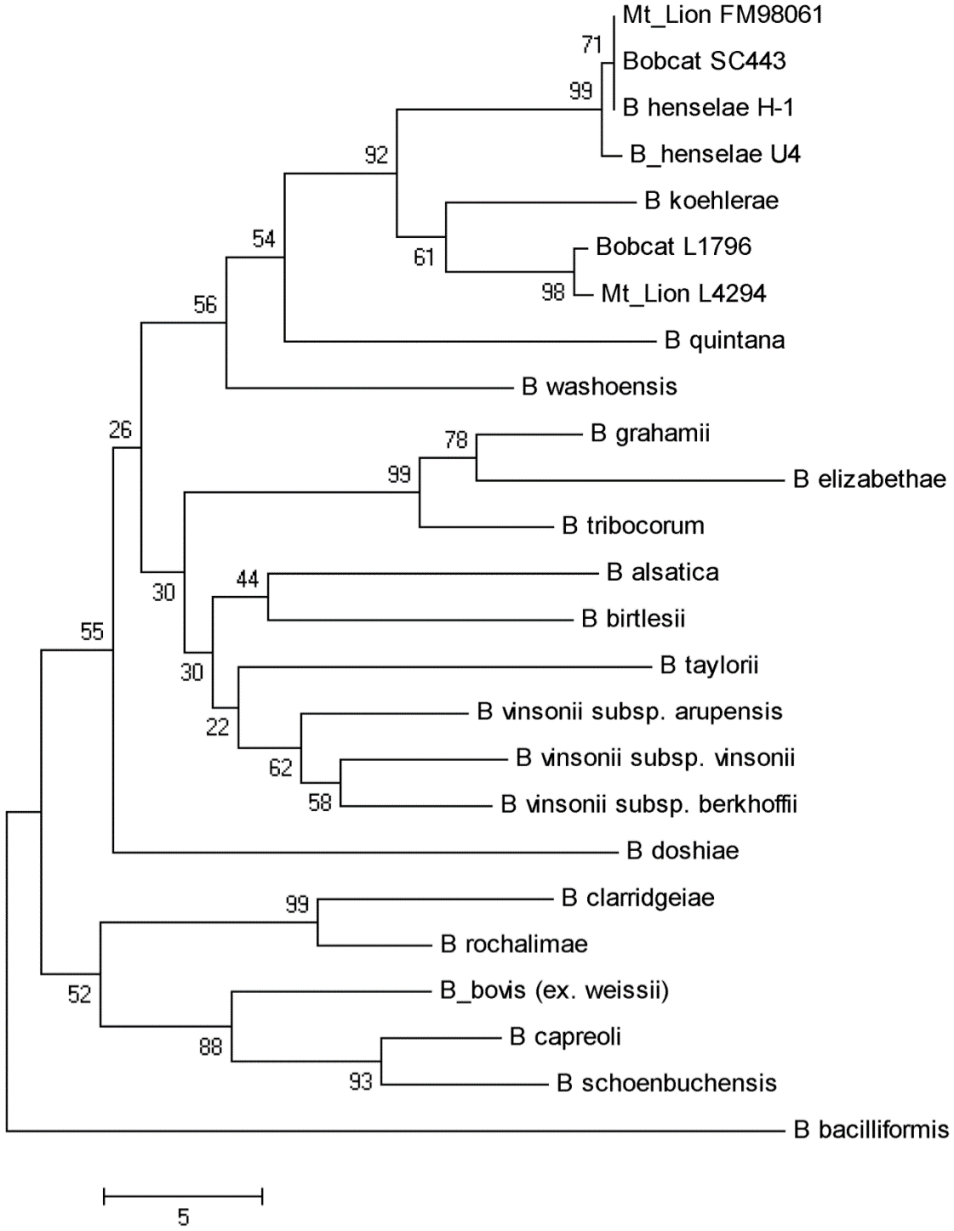
Phylogenetic relationships based on the sequence of a citrate synthase (*gltA*) gene fragment amplified from *Bartonella* species isolated from selected mountain lions and bobcats, compared with known species of the genus *Bartonella*. The phylogenetic tree was constructed from 270 bp sequences, inferred using the Neighbor-Joining method, and 1000 replicates in the bootstrap test. Scale bar indicates 5 substitutions per nucleotide position.

The sequence of 379 bp in the 3’-end of the *gltA* gene was determined for the strains isolated from mountain lions and for the strains isolated from bobcats (Fig 3 includes, as an example, two representative mountain lion strains [L-42-94, FM98061] and two representative bobcat strains [SC443, L-17-96]). Mountain lion isolates L-42-94, L-39-97 and L-27-96 (GenBank Accession Numbers: KF246521, KF246522 and KF246523, respectively [Table 6]) matched 100%, and bobcat isolates L-08-96, L-17-96, DS08, L-10-97, L-11-97 and DS507 (GenBank Accession Numbers: KF246529, KF246528, KF246524, KF246527, KF246526, and KF246525, respectively) matched 100% (Table 7). The alignment between the mountain lion and bobcat isolate sequences showed one bp substitution (a C in bobcat isolates and a T in mountain lion isolates), which translates to a synonymous amino acid. The *gltA* sequence of bobcat Sc443 (GenBank #KF466249) was 100% identical to *B*. *henselae*. The *gltA* sequences of mountain lion FM98061 (GenBank #KF466248) and bobcat isolate L-17-96 shared 94% homology with *B*. *henselae* (16 to 17 bp difference) and 95.6% homology with *B*. *koehlerae* (10 to 11 bp difference). All other *gltA* gene sequences available in the EMBL-GenBank database had a lower percent similarity (<93% similarity) when compared to these bobcat and mountain lion *gltA* sequences. When the corresponding, predicted amino acid sequences of the *gltA* genes were compared, all wild felid sequences were identical to each other, and four amino acids among 120 (3%) remained different from the amino acid sequence of the *B*.*henselae gltA* gene.

**Table 6:** GenBank Accession Numbers.

Animal ID	*gltA*	*ftsZ*	*rpoB*	16–23 ITS
**L-42-94**	KF246521	KF246530	KF246539	KF437493
**L-39-97**	KF246522	KF246531	KF246540	KF437494
**L-27-96**	KF246523	KF246532	KF246541	KF437495
**DS08**	KF246524	KF246533	KF246542	KF437496
**DS507**	KF246525	KF246534	KF246543	KF437497
**L-11-97**	KF246526	KF246535	KF246544	KF437498
**L-10-97**	KF246527	KF246536	KF246545	KF437499
**L-17-96**	KF246528	KF246537	KF246546	KF437500
**L-08-96**	KF246529	KF246538	KF246547	KF437501
**FM98061**	KF466248	KF466252	KF466254	KF466250
**SC443**	KF466249	KF466253	KF466255	KF466251

**Table 7:** DNA relatedness of L-42-94 mountain lion isolate with other *Bartonella* species.

Source of unlabeled DNA	L-42-94			*B*. *koehlerae* ATCC 700693T^a^			*B*. *henselae* ATCC 49882T			*B*. *henselae* G6486		
	55°C	D^b^	70°C	55°C	D	70°C	55°C	D	70°C	55°C	D	70°C
**L-42-94**	100	0.0	100	87	6.7	55	75	7.0	43	–^c^	–	–
***B*. *koehlerae* ATCC 700693T**	75	5.5	59	100	0.0	100	65	5.5	41	79	6.0	61
***B*. *henselae* ATCC 49882T**	77	6.0	48	84	6.5	51	100	0.0	100	100	0.5	100
***B*. *henselae* G6486**	–	–	–	78	6.0	54	83	0.5	90	100	0.0	100
***B*. *henselae* G6529**	–	–	–	68	5.5	54	85	0.5	88	–	–	–
***B*. *henselae* G8378**	–	–	–	79	6.5	60	92	0.0	94	–	–	–
***B*. *henselae* 88–712**	–	–	–	83	7.0	62	100	0.5	100	–	–	–
***B*. *henselae* G5691**	–	–	–	84	5.0	48	95	0.5	95	–	–	–
***B*. *quintana* ATCC VR-358T**	58	–	–	56	9.5	31	46	8.5	20	57	9.0	34
***B*. *vinsonii* ATCC VR-152T**	65	10.0	27	55	10.5	29	50	8.0	26	63	8.5	43
***B*. *vinsonii* subsp. *berkhoffii* ATCC 51672T**	–	–	–	46	12.0	–	42	10.5	–	–	–	–
***B*. *elizabethae* ATCC 49927T**	52	–	–	53	13.0	21	46	11.0	21	–	–	–
***B*. *clarridgeiae* Big Blackie**	48	10.0	32	–	–	–	–	–	–	–	–	–
***B*. *bacilliformis* KC 584**	35	–	–									
***B*. *bacilliformis* ATCC 35685T**	–	–	–	34	12.5	–	33	11.0	–	–	–	–

^a^ T, type strain

^b^ D, divergence within related sequences, calculated to the nearest 0.5%

^c^–, not done

**Fig 4:**
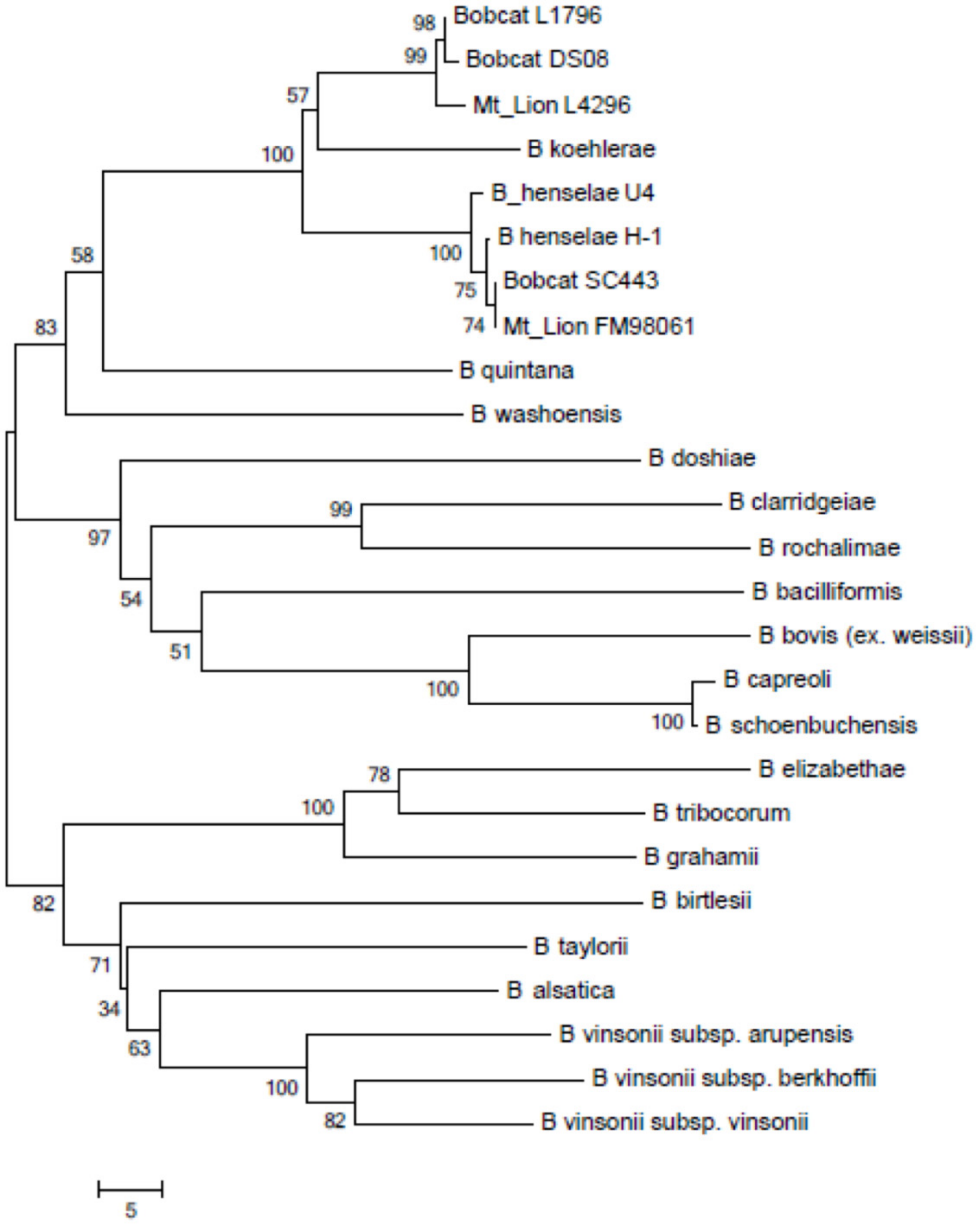
Phylogenetic relationships based on the sequence of an *rpoB* gene fragment amplified from *Bartonella* species isolated from selected mountain lions and bobcats, compared with known species of the genus *Bartonella*. The phylogenetic tree was constructed from 771 bp sequences, inferred using the Neighbor-Joining method, and 1000 replicates in the bootstrap test. Scale bar indicates 5 substitutions per nucleotide position.

The sequence of 893 bp in the 3’-end of the *rpoB* gene also was determined for the strains isolated from mountain lions and from bobcats (Fig 4 includes, as an example, two representative mountain lion strains [L-42-94, FM98061] and three bobcat strains [SC443, DS08 and L-17-96]). The *rpoB* gene sequences of bobcat Sc443 (GenBank #KF466253) and mountain lion FM98061 (GenBank #KF466252) were 100% identical and 99.6% identical to *B*. *henselae* (1 bp difference), but translated to the same amino acid as *B*. *henselae rpoB*. The sequences of the other mountain lion isolates (GenBank #KF246539 [L-42-94]; #KF246540 [L-39-97]; and #KF246541 [L-27-96]) matched 100% to each other, as well as most bobcat isolates (GenBank #KF246542 [DS507]; #KF246543 [L-11-97]; #KF246544 [L-10-97]; #KF246545 [L-17-96]; and #KF246546 [L-08-96]) all matched 100%, but shared a 96.6 to 97% homology with *B*. *henselae* (23 to 26 bp difference) and 96.5% homology with *B*. *koehlerae* (26 to 27 bp difference). However, bobcat isolate DS08 (GenBank #KF246547) had one bp substitution leading to a non-synonymous amino acid change. The alignment between the mountain lion and bobcat isolate sequences showed three bp substitutions; two of the substitutions were synonymous and the third substitution was non-synonymous but conserved (mountain lion: valine versus bobcat: alanine). All other *rpoB* gene sequences available in the EMBL-GenBank database had a lower percent similarity (< 93%).

A 935 bp fragment of the *ftsZ* gene also was analyzed. The *ftsZ* sequences from mountain lion isolates L-42-94, L-27-96 and l-39-97 (GenBank accession numbers: KF246530, KF246532 and KF246531, respectively) matched 100%, and *ftsZ* sequences from bobcat isolates L-08-96, L-17-96, DS08, L-10-97, L-11-97 and DS507 matched 100% (GenBank accession numbers: KF246538, KF246537, KF246533, KF246536, KF246535 and KF246534, respectively, Table 6). Alignment between mountain lion and bobcat isolate sequences showed two bp substitutions, which translated to a synonymous amino acid sequence. The *ftsZ* sequences for mountain lion isolate FM98061 (GenBank #KF466250) and bobcat isolate SC443 (GenBank #KF466251) matched 100%, and the *ftsZ* sequence of both isolates had one bp substitution compared to the corresponding *ftsZ* fragment in *B*. *henselae*. However, the substitution still translated to the same amino acid as *B*. *henselae ftsZ*.

Sequences for the 16S-23S ITS region from mountain lion isolates L-42-94, l-27-96 and l-39-97 matched 100% (GenBank #KF437493, KF437495, and KF437494, respectively), and sequences from bobcat isolates L-08-96, L-17-96, L-10-97, L-11-97 and DS507 matched 100% (GenBank #KF437501, KF437500, KF437499, KF437498, and KF437497, respectively, Table 6). However, bobcat isolate DS08 had one mutation compared to the other bobcat isolates. Mountain lion isolate FM98061 and bobcat isolate SC443 16S-23S ITS region sequences matched 100% (GenBank #KF466254 and KF466255, respectively). When compared to the corresponding 16S-23S ITS fragment sequence in *B*. *henselae*, the 16S-23S ITS sequences of both these isolates had three bp substitutions. Insertions and deletions (five total) in the 16S-23S ITS region sequence resulted in a fragment of: 723 bp in size for bobcat isolates L-08-96, L-17-96, L-10-97, L-11-97, DS507 and DS08; 722 bp for mountain lion isolate FM98061 and bobcat isolate SC443; and 721 bp for mountain lion isolates L-42-94, L-27-96, L-39-97.

### Phylogenetic analysis based on concatenated sequences of gene fragments

**Fig 5:**
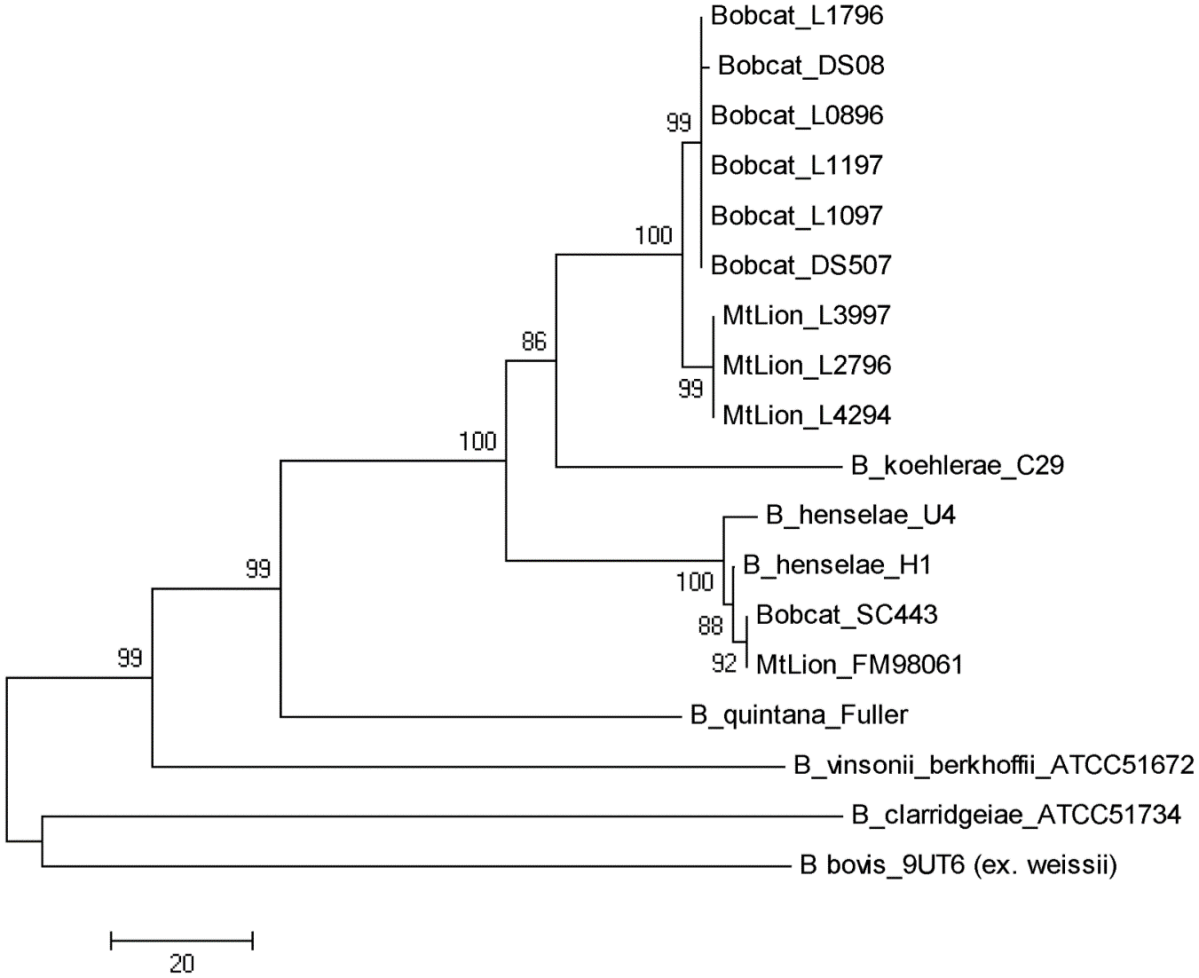
Phylogenetic relationships based on the concatenated sequences of *gltA*, *ftsZ*, and *rpoB* gene fragments and the 16S-23S ITS region of the four mountain lion isolates and seven bobcat isolates, compared with known species of the genus *Bartonella*. The phylogenetic tree was constructed from 2163 bp sequences, inferred using the Neighbor-Joining method, and 1000 replicates in the bootstrap test. Scale bar indicates 20 substitutions per nucleotide position.

Analysis of concatenated fragments of the *gltA*, *ftsZ* and *rpoB* genes and the 16S-23S rRNA ITS showed that three of the four mountain lion isolates were identical and clustered together. The six bobcat isolates were identical and were most closely related to, but distinct from, these three mountain lion isolates (Fig 5). They branched on a tree that also included *B*. *koehlerae* and *B*. *henselae*. As observed by PCR-RFLP, the two isolates from mountain lion FM98061 and bobcat SC443 were clustered with *B*. *henselae*.

### DNA-DNA hybridization studies

Levels of DNA relatedness were determined by hybridizing labeled DNA from the mountain lion isolate L-42-94, the type strain of *B*. *koehlerae* (ATCC 700693), the type strain of *B*. *henselae* (ATCC 49882), and an additional strain of *B*. *henselae* (G6486) (Table 7, top horizontal row) with unlabeled DNA from L-42-94 and 14 *Bartonella* species or subspecies (Table 7, far left column). Labeled DNA from the type strain of *B*. *koehlerae* had an average relatedness to the mountain lion strain L-42-94 of 87% with 6.5% divergence in optimal DNA reassociation reactions at 55°C. In stringent DNA reassociation reactions at 70°C, they were 55% related. Labeled DNA from the type strain of *B*. *henselae* showed a relatedness to the mountain lion *Bartonella* isolate L-42-94 of 75% at 55°C with 7.0% divergence, and 43% at 70°C.

Using labeled L-42-94 DNA hybridized with unlabeled DNA from the type strain of *B*. *koehlerae*, the mountain lion L-42-94 DNA showed a relatedness of 75% at 55°C with 5.5% divergence, and of 59% at 70°C (Table 7). Using labeled L-42-94 DNA hybridized with unlabeled DNA from the type strain of *B*. *henselae*, L-42-94 showed a relatedness of 77% at 55°C with 6.0% divergence, and of 48% at 70°C. Based on DNA hybridization studies, our isolate L-42-94 was most closely related to *B*. *koehlerae*: range and relatedness of L-42-94 to the type strain of *B*. *koehlerae* was 75% to 87%, with 5.5% to 6.5% divergence at 55°C; range and relatedness of L-42-94 to the type strain of *B*. *henselae* was 75% to 77%, with 6.0% to 7.0% divergence at 55°C. The relatedness of L-42-94 to type strains *B*. *quintana* ATCC VR-358, *B*. *vinsonii* ATCC VR-152, and *B*. *elizabethae* ATCC 49927 ranged from 52% to 68% at 55°C. DNA from L-42-94 was least related (35% to 48%) to *B*. *clarridgeiae* strain “Big Blackie” and *B*. *bacilliformis* strain KC 584 by DNA-DNA hybridization studies.

## Discussion

Numerous studies have demonstrated the high prevalence of prolonged *B*. *henselae* bacteremia in domestic cats of northern California and the subsequent risk of cat scratch disease in their owners [1, 5, 18]. In addition, it has been well documented that infectious organisms can be readily transferred from wild mammals to domestic pets or humans, either directly, or via an arthropod vector [2]. We therefore sought to determine whether wild felids in northern California provide a bacteremic reservoir for *Bartonella*, and if so, to define the infecting *Bartonella* species. In this study, we report the isolation of *B*. *henselae* and the first isolation of two new *B*. *koehlerae* subspecies from free-ranging wild felids in North America.

Evidence of *Bartonella* infection in wild felids in northern California was first reported in bobcats from Marin County, CA, when serum testing revealed seroreactivity to *B*. *henselae* antigen in 74% of 25 bobcats [30]. More recently, *Bartonella* DNA was detected in strongly seropositive California mountain lions, and sequences of amplified DNA fragments were reported to be identical to *B*. *henselae* [12]. *Bartonella* spp. also have been found in African felids. Kelly *et al*. [31] reported the isolation of *B*. *henselae* from an African cheetah from Zimbabwe. Similarly, *B*. *henselae* and an unidentified *Bartonella* strain with a PCR-RFLP profile similar to *B*. *koehlerae* were isolated from African free-ranging lions (*Panthera leo*) from Kruger National Park, South Africa [32]. Moreover, *B*. *henselae* was isolated from semi-captive lions from three ranches in the Free State Province, South Africa [33].

In our study, we found a prevalence of *Bartonella* bacteremia in 29% and 37% of California mountain lions and bobcats, respectively, providing further evidence that *Bartonella* bacteremia is common in free-ranging wild felids. Such a prevalence of *Bartonella* bacteremia is similar to what has been reported for *B*. *henselae* in domestic cats from northern California [5, 18], as well as for *Bartonella* spp. from other wild carnivores in northern California, including 42% of 53 gray foxes (*Urocyon cinereoargenteus*) infected with *B*. *rochalimae* [34], 28% of 109 coyotes (*Canis latrans*) infected with *B*. *vinsonii* subsp. *berkhoffii* [19] in central California, and 26% of 42 raccoons (*Procyon lotor*) bacteremic with *B*. *rochalimae* [35]. Domestic cats are the only previously known, culture-positive reservoir of *B*. *henselae*, *B*. *clarridgeiae* and *B*. *koehlerae* [36]. In this study, we demonstrated that wild felids from California are likely the natural reservoirs of several *Bartonella* species, and they can be long-term carriers of these *Bartonella* spp, as previously documented in domestic cats [3, 36].

The strains isolated from mountain lions and bobcats were morphologically similar to *B*. *henselae*, but were not apparent on blood agar until two weeks after plating. In contrast, after culturing blood of infected domestic cats, *B*. *henselae* colonies are usually visible within a few days. This characteristic of two weeks’ incubation time to appearance of colonies also was observed in domestic specific-pathogen free kittens experimentally infected with one of the mountain lion isolates [37].

The biochemical profiles of mountain lion and bobcat isolates were consistent with the general profile observed for *Bartonella* species. RFLP analysis of a PCR-amplified *gltA* gene fragment indicated that two endonucleases, *Taq*I and *Aci*I, are useful to specifically distinguish the mountain lion and bobcat strains from each another and from *B*. *henselae* and *B*. *koehlerae*. By pulsed field gel electrophoresis and sequencing of the 16S rRNA gene, it appeared that these wild felid isolates are different from any isolate found in domestic cats. PFGE band patterns were identical for three of the four mountain lion isolates but differed from the bobcat isolates. However, one bobcat and one mountain lion were infected not with a new *Bartonella* subspecies, but with *B*. *henselae*, suggesting that diverse *Bartonella* species can infect these two mammalian reservoir species.

DNA-DNA hybridization remains a reliable method for defining a species. It has been recommended that a species consists of strains whose DNA is ≥70% related at optimal re-association conditions, ≥55% related under stringent DNA re-association conditions, and also, whose DNA contains ≤5% divergence within related sequences [29, 38]. Labeled DNA from L-42-94 showed relatedness to the type strain of *B*. *koehlerae* of 75% (with 5.5% divergence) at 55°C, and 59% at 70°C. The percent DNA relatedness was higher in reciprocal reactions at 55°C but not at 70°C, where labeled DNA from the type strain of *B*. *koehlerae* showed a relatedness to the *Bartonella* mountain lion isolate L-42-94 of 87% (with 6.5% divergence) at 55°C, and 55% at 70°C. Relatedness values that demonstrate non-reciprocity have been identified in strains from many genera, and this finding emphasizes the importance of performing reciprocal DNA relatedness when studying strains that are close to the species definition. Non-reciprocity can be observed when the genomes of the two species being compared are of different sizes [4]. For relatedness under stringent conditions, the mountain lion isolate L-42-94 fulfills the strict criteria for belonging to the same species as *B*. *koehlerae*. However, for divergence, L-42-94 does not fulfill the strict criteria for belonging to the same species as *B*. *koehlerae*, and thus, L-42-94 meets the criteria for being a subspecies of *B*. *koehlerae*.

In natural conditions, it appears that free-ranging wild cats can be infected with *Bartonella* species found in domestic cats, as demonstrated by the isolation of *B*. *henselae* genotype II from a very young bobcat and a young mountain lion. The juvenile mountain lion was held in the CDFW facility; therefore, it is difficult to determine if this specific strain was acquired when this animal was kept in captivity or prior to its rescue from the wild. The strain from the bobcat could have been acquired by exposure to fleas from domestic cats, because stray cats are common in the area where this young bobcat was found (Daren Simpson, personal communication). These results clearly show that *B*. *henselae* can naturally infect both domestic cats and wild free-ranging felids.

Of note, the novel strains isolated from the mountain lions and bobcats appear to be highly adapted to their specific host, and could have evolved and adapted from a common *Bartonella* ancestor within these specific populations. Interestingly, these isolates are phylogenetically intermediate between *B*. *henselae* and *B*. *koehlerae*, two species for which domestic cats are the main known reservoir. Further studies should be conducted to investigate the evolution of these different *Bartonella* species among domestic and wild felids. To date, neither of these new *Bartonella* subspecies has been isolated from domestic cats. However, we previously demonstrated [37] that domestic cats represent a permissive host for experimental infection with a mountain lion *Bartonella* isolate (strain L-42-94). We also found that there was no cross-protection between this strain and *Bartonella* strains that infect domestic felines [37]. We were not able to identify any natural infection of these California free-ranging wild felids with *B*. *clarridgeiae* or *B*. *koehlerae*, the two *Bartonella* species that are less commonly isolated from domestic cats in the western USA [1]. The small sample size we tested may not have been sufficient for detection of these two *Bartonella* species from wild-ranging felids.

The zoonotic potential of these novel *Bartonella* isolates is unknown. Mountain lion and bobcat attacks on humans are quite rare. However, over the past several decades, the incidence of mountain lion attacks on people has been slowly increasing, with more events occurring during the past 22 years than in the last century [39]. There were 50 documented attacks on children with a 25% fatality rate out of 83 documented mountain lion attacks over the last 100 years [39]. Most children were not alone at the time of the attack (92%), and in many instances adult supervision was present or nearby. Severe head and neck lacerations along with puncture wounds were the most common injuries. Disease transmission in such encounters is also possible, as illustrated by two people who developed *Pasteurella* infection after being attacked. Additionally, two mountain lions implicated in attacks on humans were confirmed to be rabid [39]. A case of *Pasteurella multocida* infection also was acquired following a bite by a pet mountain lion [40]. No evidence of cat scratch disease was observed in this case, but the pet had previously been declawed. Potential exposure to ectoparasites infected with *Bartonella*, especially fleas infesting a mountain lion or a bobcat, could be a risk factor for humans, because fleas have been shown to effectively transmit *B*. *henselae* between cats [41]. Additional research is needed to definitively identify the vector and mode of transmission of *Bartonella* between wild felids.

It is most likely that the northern California bobcats and mountain lions are naturally infected with novel *Bartonella* species or subspecies that are specific to each felid species, and in addition, sometimes can be infected with *B*. *henselae*. This is based on our findings that a) we were able to differentiate the mountain lion isolates from the bobcat isolates by RFLP, PFGE, and partial sequencing of at least four different genes; but b) we could not differentiate between isolates from the same reservoir species; and c) these isolates from bobcats and mountain lions were closely related to, but distinct from, the reference *B*. *henselae* and *B*. *koehlerae* strains.

The criteria established by La Scola et al. [42] to define a new *Bartonella* species indicate that the percentage of similarity should be ≤96% for *gltA* and ≤95.6% for *rpoB*. However, with these two *Bartonella* isolates, the similarity values were below the threshold for *gltA* (94%-95.6%) gene, but above the threshold for *rpoB* (96.6% to 97%) gene, and thus these two novel *Bartonella* isolates are located phylogenetically between *B*. *henselae* and *B*. *koehlerae*. Because DNA-DNA hybridization reveals that L-42-94 is more closely related to *B*. *koehlerae* than *B*. *henselae*, we propose creating two new subspecies of *B*. *koehlerae*: *B*. *koehlerae* subsp. *boulouisii* and *B*. *koehlerae* subsp. *bothieri*, for which mountain lions and bobcats are the respective natural reservoirs. The identification of a cognate and unique mammalian reservoir for each of these two novel subspecies also should be noted.

### Emendation of the description of *Bartonella koehlerae*

*Bartonella koehlerae* (Droz S, Chi B, Horn E, Steigerwalt AG, Whitney AM, Brenner DJ. 1999). The characteristics of this taxon are the same as those described for the genus [43] and those described by Droz *et al*. [4] for the species. The species now contains three subspecies: one that was isolated previously from domestic cats and humans [4, 44, 45]; another that was isolated only from mountain lions (*Puma concolor*); and the third that was isolated only from bobcats (*Lynx rufus*).

### Description of *Bartonella koehlerae* subsp. *koehlerae* subsp. nov.

*Bartonella koehlerae* subsp. *koehlerae* subsp. nov. (koeh′ ler. ae. N. L. fem. adj. *koehlerae*. The type strain C-29 (ATCC 700693) was recovered from the blood of a healthy kitten during a prevalence study of *B*. *henselae* in domestic cats in the greater San Francisco Bay area, northern California [4].

### Description of *Bartonella koehlerae* subsp. *boulouisii* subsp. nov.

*Bartonella koehlerae* subsp. *boulouisii* (bou. lou. is′ i. i. N. L. gen. masc. n. *boulouisii* of Boulouis, in honor of Henri-Jean Boulouis, a veterinary microbiologist and professor at the School of Veterinary Medicine in Maisons-Alfort, France, whose wide interest in the field of *Bartonella* infections in animals and humans led to the isolation of several new *Bartonella* species). The main phenotypic characteristics are identical to those of the genus *Bartonella*. The strain is oxidase and catalase negative. Primary isolation from mountain lion blood occurred on HIAR agar, where the first colonies were observed after 14 days of incubation at 35°C in a candle jar (with a CO_2_-enriched environment). The code for preformed enzymes obtained in the MicroScan Rapid Anaerobe Panel is 10077640. The new *B*. *koehlerae* subspecies shows a unique PCR-RFLP pattern for the citrate synthase gene (*gltA*) and a unique PFGE pattern that differs from those of *B*. *henselae* and *B*. *koehlerae*. The type strain, L-42-94, recovered from the blood of a mountain lion (*Puma concolor*) during a prevalence study of *Bartonella* infections in wild cats in northern California, has been deposited at the ATCC, USA (ATCC BAA-2635; http://www.atcc.org/) and at the Collection of Institut Pasteur (CIP), Paris, France.

### Description of *Bartonella koehlerae* subsp. *bothieri* subsp. nov.

*B*. *koehlerae* subsp. *bothieri* (bo.thi'er.i. N.L. masc. gen. n. bothieri, of Bothier, in honor of François Bothier, a French physician and hematologist from Lyon, France, specialist of blood and blood-borne diseases). The main phenotypic characteristics are identical to those of the genus *Bartonella*. The strain is oxidase and catalase negative. Primary isolation from bobcat blood occurred on HIAR agar, where the first colonies were observed after 14 days of incubation at 35°C in a candle jar (with a CO_2_-enriched environment). The code for preformed enzymes obtained in the MicroScan Rapid Anaerobe Panel is 10477640. The new *B*. *koehlerae* subspecies shows a unique PCR-RFLP pattern for the citrate synthase gene (*gltA*) PCR-RFLP and a unique PFGE pattern, which are different from those of *B*. *henselae* and *B*. *koehlerae*. The type strain, L-17-96, recovered from the blood of a bobcat (*Lynx rufus*) during a prevalence study of *Bartonella* infections in wild cats in northern California, has been deposited at the ATCC, USA (ATCC BAA-2636; http://www.atcc.org/) and at the Collection of Institut Pasteur (CIP), Paris, France.
